# Markers of Gut Health in Small Animals: Focus on Fatty Acids and Amino Acids as Indicators of Intestinal Functionality and Microbiome Activity

**DOI:** 10.3390/ani15131927

**Published:** 2025-06-30

**Authors:** Ana I. Rey, Cristina Higueras, Patricia Olmeda, Angel Sainz, Beatriz G. Gálvez, Mar Larrosa

**Affiliations:** 1 Animal Nutrition, Department of Animal Production, Faculty of Veterinary Medicine, Complutense University of Madrid, Avda. Puerta de Hierro s/n., 28040 Madrid, Spain; 2 Department of Animal Medicine and Surgery, Faculty of Veterinary Medicine, Complutense University of Madrid, Avda. Puerta de Hierro s/n., 28040 Madrid, Spain; 3 Department of Biochemistry and Molecular Biology, Faculty of Pharmacy, Complutense University of Madrid, Plaza Ramón y Cajal s/n., 28040 Madrid, Spain; 4 Department of Nutrition and Food Science, Faculty of Pharmacy, Complutense University of Madrid, Plaza Ramón y Cajal s/n., 28040 Madrid, Spain

**Keywords:** gut health, amino acids, fatty acids, short-chain fatty acids, food-responsive enteropathy, immunosuppressant-responsive enteropathy, dogs, cats, IBD

## Abstract

Chronic inflammatory enteropathies (CIEs) in companion animals represent a group of idiopathic, immune-mediated gastrointestinal disorders in which the intestinal epithelium can be altered, affecting intestinal functionality, nutrient absorption, and microbiota composition. This review presents an overview of markers that could be used for the assessment of intestinal health, focusing extensively on functional biomarkers, with particular attention to fatty acids (including short-chain fatty acids, SCFAs) and amino acids. Studies have consistently shown reduced concentrations of SCFAs in companion animals with CIEs compared to healthy groups. These alterations occur with varying intensity depending on the type of enteropathy. Alterations in saturated, monounsaturated, and long-chain polyunsaturated fatty acids have also been reported in blood and feces, particularly in omega-3 and omega-6 derivatives, as well as in the elongase and desaturase indices responsible for endogenous synthesis. In addition, amino acids serve as precursors to key metabolites involved in mucosal immunity, oxidative stress regulation, and microbial homeostasis. In CIEs, alterations in systemic and fecal amino acid profiles have been observed, reflecting both host metabolic adaptation and microbial dysbiosis. Integrating fatty acid and amino acid profiles can help distinguish different types of enteropathies, providing additional discriminatory power for determining response to dietary treatment. Future research should aim to elucidate the causal relationships between metabolic alterations and disease pathogenesis, which could lead to novel dietary interventions targeting metabolic interactions between the microbiota and the host.

## Simple Summary

This review analyzes the role of fatty acids and amino acids as biomarkers of gut health and microbiota activity in dogs and cats with chronic inflammatory enteropathies (CIEs). Alterations in short-chain fatty acids, long-chain polyunsaturated fatty acids, and amino acid profiles are linked to malabsorption, dysbiosis, and immune dysregulation. These metabolites might help differentiate between types of CIEs and monitor the response to dietary treatment.

## 1. Introduction

In recent years, the role of the intestine in overall health has gained significant importance. This organ not only plays a crucial role in host nutrition, but also contributes to defense against infections, immune tolerance, and the production of metabolites that serve as signaling molecules in other organs, such as the brain [1]. Consequently, dysfunction of the intestine has been linked to various human diseases, including inflammatory bowel disease (IBD), cardiovascular diseases, autoimmune disorders, and degenerative neurological conditions [2].

In animals, various diseases associated with digestive system dysfunction have also been identified [3,4,5], including chronic inflammatory enteropathies (CIEs) similar to human IBD. However, the exact mechanisms underlying their development remain unclear. Potential factors such as diet and environment have been recognized [2]. In the specific case of CIEs in companion animals, first-line treatment consists of dietary modification using hypoallergenic diets (including those containing hydrolyzed proteins or novel protein sources and easily digestible ingredients) [6,7]. However, not all animals respond to dietary treatment, and the reasons why some do while others do not remain unknown. This has led to the establishment of different types of CIEs in veterinary medicine, among which the most common are those that respond to diet (FRE) and those that respond to immunosuppressants (IRE), the latter also known as IBD [6]. The need to wait for a response to dietary treatment using various diets significantly prolongs the diagnostic process, negatively impacting the animal. Additionally, invasive techniques such as endoscopy and biopsy are often required to expedite diagnosis and detect inflammatory infiltrates at the intestinal level [8]. As a result, identifying markers of digestive health is essential to achieve more accurate diagnoses and to establish appropriate treatment strategies.

**Figure 1:**
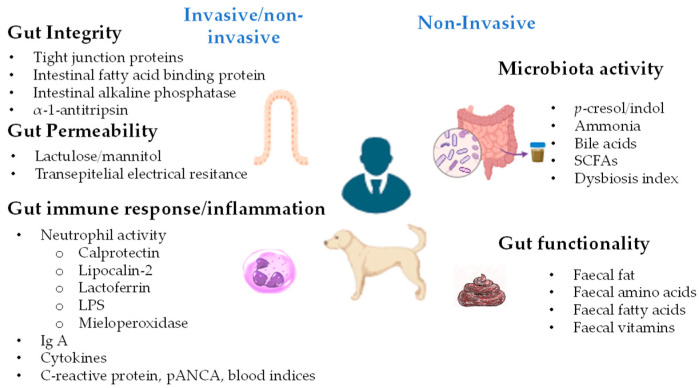
Invasive and non-invasive markers of gut health.

Before delving deeper into the study of possible markers of digestive health, it is essential to clarify what we mean by good health. According to different authors, good digestive health is associated with efficient digestion and nutrient absorption, stable microbiota, a well-maintained gastrointestinal epithelium and mucosal barrier, and a properly functioning immune response and enteric nervous system [1,9]. Therefore, based on this, digestive health could be evaluated by searching for markers that indicate the integrity of the intestinal barrier, the immune response at this level, the functionality of the digestive system, or the microbiota activity (Figure 1).

## 2. Markers for Gut Health Evaluation

### 2.1. Intestinal Barrier Integrity Indicators

Intestinal barrier integrity biomarkers enable the assessment of the intestinal mucosa’s state and its ability to maintain a selective barrier against pathogens and toxins [10]. Disruption of this barrier can result in increased intestinal permeability, translocation of luminal content between cells or through the cells [11], systemic inflammation, and dysbiosis [12].

To assess gut permeability, there are minimally invasive methods based on the oral ingestion of specific molecules and their detection in urine or blood. The number of studies performed in small animals is still limited. Thus, lactulose (L) (a large disaccharide) is only absorbed when intestinal permeability is increased (paracellular route); whereas mannitol (M) (a small monosaccharide) is absorbed through an intact intestinal epithelium (transcellular route) [13]. Consequently, a higher ratio of L/M suggests impaired barrier function [14,15]. There is also the possibility of using a multi-sugar test (sucrose, lactulose, L-rhamnose, erythritol, sucralose) to assess permeability across different gut regions [16], but this requires urine collection for up to 24 h, which may reduce compliance. Also, these methods could be affected by renal function, hydration status, and gastric emptying time [17], and it has been reported that breed and some other environmental factors should be taken into account [14].

Moreover, several circulating biomarkers are used to assess intestinal barrier dysfunction. Hence, higher serum zonulin levels are associated with gut barrier dysfunction in patients with celiac disease [18], since this protein modulates tight junctions [18]. Its circulating levels have also been shown to change after probiotic enema treatment [19] or probiotic treatment [20] in dogs. However, other authors did not differentiate between dogs with chronic enteropathies and control dogs using commercial canine zonulin in serum [21] or fecal samples [22].

Also, citrulline, a non-protein amino acid produced by enterocytes, has been found to decrease in cases of enterocyte loss [23]. Thus, a reduction of more than 50% in plasma L-citrulline levels during the treatment period has been strongly correlated with histopathological changes in dogs [24]. Also, in dogs, Xu et al. [25] found a decrease in plasma citrulline and an inverse correlation of this compound with the Canine Chronic Enteropathy Clinical Activity Index (CCECAI). However, other authors did not find a correlation of serum citrulline with the Chronic Inflammatory Bowel Disease Activity Index (CIBDAI) or serum albumin concentration, and it was not associated with efficacy of treatment in dogs with CIEs [26]. Fatty acid binding proteins (FABPs) have also been found to be elevated in intestinal ischemia [27], IBD, and acute intestinal injury in infants [28], since these I-FABP are released into the bloodstream when enterocytes are damaged. However, in dogs, some authors did not find changes after gut health improvement [20].

Alpha-1-antitrypsin is also a serum protein secreted in the liver that protects tissues from the proteolytic activity of immune cells [29]. This protein leaks into the gut when permeability increases; therefore, higher fecal levels have been observed in Crohn’s disease (CD) and enteric dysfunction [30,31]. This is considered a reliable fecal marker since it is a proteolysis-resistant glycoprotein to enzymatic degradation [32]. It has also been reported to be useful for early detection of gastrointestinal protein loss in dogs with protein-losing enteropathy (PLE) [33] using serum or fecal samples, and with caution in the interpretation in corticosteroid-treated dogs [31].

Another compound that plays an interesting role in intestinal homeostasis is intestinal alkaline phosphatase (IAP). This brush border enzyme has the ability to detoxify some microbial ligands such as lipopolysaccharides, maintaining a healthy gastrointestinal tract [34]. Thus, it has been found to function as a gut mucosal defense factor limiting the translocation of gut bacteria to lymph nodes and preserving gut microbial homeostasis [35]. Dogs with CIEs have shown impaired IAP activity in fecal samples, but its role in pathogenesis is not clear [36].

In summary, most intestinal integrity indicator techniques are invasive, except in certain cases where fecal measurement is possible. Although they appear to be useful in humans, there is some disagreement among authors regarding their findings in dogs, with variations depending on certain animal-specific or environmental factors.

### 2.2. Indicators of Gut Immune Response

Other indirect markers of intestinal inflammation rather than permeability itself include calprotectin, calgranulin (or S100A12), and myeloperoxidase (MPO). These enzymes are released by activated mononuclear cells, mainly neutrophils, in response to intestinal barrier damage. Elevated levels in stool indicate intestinal inflammation and epithelial damage; therefore, these are useful in differentiating IBD from functional gastrointestinal disorders [37]. Thus, calprotectin has been found to increase in dogs with CIEs [38], with levels correlating with histopathologic findings and clinical outcome [39], and might be used as an indicator of disease severity [39,40,41]. Similarly, fecal MPO levels has been found to be increased in dogs with CIEs, showing similar correlations to those observed for calprotectin [42]. Increased mucosal expression of calgranulin has also been documented in dogs with chronic intestinal inflammation, therefore making it a valuable biomarker for disease detection, severity grading, and treatment prognosis [43,44].

Lipopolysaccharides (LPS) are glycolipids of the outer membranes of Gram-negative bacteria that enter circulation when gut permeability is increased [45]. Thus, their detection in blood signals bacterial translocation from the intestinal lumen, which can lead to systemic inflammation and endotoxemia [46]. However, LPS detection may originate from sources other than the gut (e.g., infections) [45]. In dogs, dietary supplementation with probiotics lowered the serum LPS levels as an indicator of improved gut health [20].

Some antimicrobial and protective proteins, such as lactoferrin and lipocalin-2, protect the gut epithelium and help in regulation of microbial populations [47,48]. Thus, lactoferrin, an iron-binding glycoprotein with antimicrobial and immune-modulatory properties, indicates gut inflammation and neutrophil activation when it is elevated [49]. This compound has the ability to both sequester iron and to direct reactive oxygen intermediates to diminish damage due to inflammatory response [49]. It can also be used to differentiate canine infectious and non-infectious diarrhea [50]. Likewise, lipocalin-2, an innate immune protein secreted by neutrophils and epithelial cells, is elevated in inflammatory conditions and gut dysbiosis [48].

On the other hand, secretory immunoglobulin A (IgA) is a key mucosal immune defense antibody that can be found in feces. High levels indicate an active immune response, while low levels suggest compromised mucosal immunity. Therefore, decreased IgA has been observed in fecal samples of dogs with CIEs [51], similar to the fecal IgA deficiency in humans with chronic gastrointestinal disease [52]; whereas an IgA increase has been found in feces of dogs fed prebiotics [53]. However, their value for clinical application is not clear, since their levels can be influenced by breed or age of the dog, as well as fecal characteristics [54].

To assess immune response and inflammation, many authors suggest the use of cytokines. These can be measured in blood, feces, and gut tissues samples. In general, elevated pro-inflammatory cytokines (IL-6, IL-12, IL-23, TNF-α) indicate gut inflammation, while IL-10 suggests immune regulation [55]. In dogs with IBD, some authors have observed higher mRNA expression levels of cytokines IL-2, IL-4, IL-5, IL-8, IL-12 (p40), IFN-γ, TNF-α, and TGF-β compared to healthy subjects [56,57]. However, other studies have found that only IL12 mRNA expression consistently increased in small-intestinal IBD, whereas IBD colitis lacked consistent patterns of expression [58].

C-reactive protein (CRP) is a positive acute-phase protein that increases in response to inflammation and tissue damage in different scenarios [59,60]. It has been demonstrated that its serum levels increase in dogs with CIEs, especially in those with a more severe condition, such as a higher clinical activity index score [61], or in cases of protein-losing enteropathy (PLE) [62,63]. Serum CRP may therefore be useful in dogs with CIEs for assessing disease severity and monitoring treatment response, as it has been shown to decrease with favorable disease progression [64]. However, due to its low specificity, CRP alone may not be sufficiently representative, and its use in combination with other biomarkers would be recommended [59].

Perinuclear anti-neutrophilic cytoplasmatic antibodies (pANCA) are serum antibodies associated with autoimmune diseases in dogs and humans [7,65]. Some studies have demonstrated that pANCA levels are elevated in dogs with food-responsive enteropathy (FRE), and that they could also be useful biomarkers for the early diagnosis of PLE in specific breeds [8,66,67]. However, elevated serum levels of pANCA are not only found in dogs with CIEs but also in other diseases [7].

More recently, certain blood indices, such as the platelet-to-lymphocyte ratio (PLR), neutrophil-to-lymphocyte ratio (NLR), and systemic immune-inflammation index (SII), which involves platelet, neutrophil, and lymphocyte counts, have been proposed as markers of systemic inflammation and may therefore assist in distinguishing between FRE and immunosuppressant-responsive enteropathy (IRE) dogs. The PLR index has been reported to be elevated in IRE dogs compared to healthy controls [68,69], similar to findings in humans with IBD [70,71]. Likewise, significant elevations in both NLR and SII have been observed in IRE dogs relative to controls [68,69]. Moreover, differences in NLR between IRE and FRE dogs have been reported [72], and Benvenuti et al. [73] demonstrated a correlation between NLR and the CCECAI.

Compared to markers of intestinal integrity, these biomarkers provide insights into disease severity, have the ability to differentiate CIEs from other gastrointestinal disorders and the potential for monitoring disease progression. However, challenges include variability in marker levels due to factors such as breed, age, and diet, as well as the low specificity of some markers, such as CRP. While some markers offer promising diagnostic value, further research is required to confirm their clinical relevance.

### 2.3. Indicators of Gut Microbiota Activity

Most of the techniques indicated so far range from mildly invasive, in the case of those requiring the study of circulating markers or markers that can be measured in feces, to more severe, when intestinal samples are required (histological evaluation). Due to the growing trend toward the use of non-invasive samples that respect animal welfare and are also easy to collect, the study of the microbiota in feces is becoming more common. However, these are complex techniques that require specific knowledge for their evaluation, making the indirect study of microbial activity more accessible.

The canine fecal dysbiosis index (DI) assesses alterations in the intestinal microbiota, with a reported sensitivity of 74% and specificity of 95% [74]. It is based on quantitative PCR detection of eight bacterial groups commonly altered in dogs with CIEs. Among these, *Fusobacterium* spp., *Fecalibacterium* spp., *Turicibacter* spp., *Blautia* spp., and *Peptacetobacter hiranonis* are typically decreased, whereas *Escherichia coli* and *Streptococcus* spp., are usually increased [74,75,76,77]. Total bacterial counts are also measured. DI values below 0 generally indicate a normal microbiota, values between 0 and 2 suggest minor shifts without clear dysbiosis, and values above 2 indicate significant dysbiosis [74]. Dogs with CIEs consistently exhibit higher DI values compared to healthy dogs. Notably, DI often remains elevated even after clinical remission or short-term treatment, suggesting that microbial dysbiosis may persist despite resolution of clinical signs [78,79,80,81].

Other indirect indicators of microbial dysbiosis are related to increased production of hydrogen sulfide, ammonia, or secondary bile salts [82]. Furthermore, bacterial alterations can modify the metabolism of aromatic amino acids with increased production of phenylalanine or tyrosine derivatives (p-cresol) or tryptophan derivatives (indole), which may contribute to the development of IBD [82]. Thus, in models of induced colitis in rats [83] and mice [84], some microbial metabolites, such as cresol-sulfate, p-cresol glucuronic, or the bile acid 12-hydroxy-3-oxocolladienic acid in urine, have been identified.

In particular, bile acid metabolism is gaining interest in dogs with chronic enteropathies. Blake et al. [85] found that dogs with gastrointestinal disease had lower concentrations of secondary bile acids in fecal samples than healthy dogs, which could be related to dysbiosis, since secondary bile acids can be synthesized from primary bile acids by the intestinal microbiota. However, primary bile acids are synthesized in the liver from cholesterol and subsequently conjugated with the amino acids glycine and taurine to be released during fat digestion, most of these being reabsorbed [86]. This alteration in secondary bile acid synthesis can have various effects on the host, as they have been associated with anti-inflammatory or immune-regulating effects [87]. Other authors also found altered bile acids metabolism in dogs with idiopathic inflammatory bowel disease [88]. However, there is still a long way to go when it comes to studying bile acids in dogs to identify different types of chronic enteropathies.

One of the most studied compounds related to microbiome activity and dysbiosis phenomena are short-chain fatty acids (SCFAs). SCFAs are products obtained by the fermentative action of microorganisms in the large intestine on dietary fiber or other compounds that escape digestion by the host, such as peptides or proteins [89]. The majority are acetic (C2), propionic (C3), and butyric (C4), while from the branched-chain amino acids (valine, isoleucine and leucine), the volatile branched-chain fatty acids isobutyrate, 2-methylbutyrate, and isovalerate are obtained [90].

These compounds are key microbial metabolites involved in gut homeostasis and immune modulation. In particular, propionic acid has a high capacity to inhibit the growth of several pathogenic bacteria (Gram-positive or negative) as well as anti-inflammatory effects [91], whereas butyrate is the primary fuel source for the colonocyte [92]. This SCFA has also interesting protective effects on the intestinal mucosal barrier integrity, and, in addition, through interactions with the G protein-coupled receptors (GPCRs), has shown immunomodulatory effects [92]. Consequently, alterations in SCFA profiles have been widely associated with CIEs in dogs and cats (Table 1), as well as IBD (Crohn’s disease: CD, and ulcerative colitis: UC) in humans. Therefore, their determination could be an indicator of microbial activity. Thus, several studies have characterized fecal, urinary, and plasma SCFA concentrations in diseased and healthy subjects, providing insight into potential microbial imbalances and metabolic dysfunctions. In this sense, one of the first studies that analyzed fecal SCFA concentrations in dogs with Inflammatory Intestinal Disease found no significant differences between affected and control groups, although in this study, an inverse relationship was observed between the CCECAI and the total SCFAs [25]. In contrast, Minamoto et al. [76] investigated 73 dogs with CIEs and 49 healthy controls, reporting a decrease in acetic acid (C2), propionic acid (C3), and total SCFAs in diseased animals. Moreover, these authors did not find differences in fecal concentrations of any of the SCFAs between dogs receiving medical treatment (antibiotic or immunosuppressive agents) and those that did not. Higueras et al. [93] further refined this analysis by focusing on food-responsive enteropathy (FRE), identifying a significant reduction in C2 and C3, as well as branched-chain fatty acids isobutyric acid (iC4) and isovaleric acid (iC5), and a negative correlation between C3 and the CIBDAI was observed. Kaga et al. [57] also confirmed lower C2 and C3 levels in IBD-affected dogs, reinforcing the role of SCFAs in gut inflammation. More recently, Higueras et al. [94] investigated different types of chronic enteropathies: FRE and IRE. They observed a decrease in C2, C3, and total SCFAs in IRE cases, similar to previous findings in CIEs, while FRE dogs showed an intermediate pattern with similarities to the control group. This study suggests that SCFA depletion may be linked to immune-mediated inflammation and could serve as a biomarker for differentiating IRE from other enteropathies. On the other hand, Miller et al. [95] studied cats diagnosed with IBD, T-phenotype small cell lymphoma, and unidentified enteropathies, comparing their SCFA profiles to those of 13 healthy felines. Their results showed a notable decrease in C3 and iC4 in diseased cats. However, an increase in butyric acid (C4) and total SCFAs was observed, which contrasts with canine and human studies that typically report SCFA depletion. This discrepancy may reflect species-specific differences in microbial metabolism or host–microbiota interactions in the feline gut. The fact that these compounds play important roles in the body raises the possibility of designing more personalized diets in companion animals. Thus, it has been reported that an increase in prebiotics in the diet produces a quadratic increase in C2, C3, and C4 in feces [96].

Supporting the results observed in companion animals, several human studies have reported SCFA deficiencies in fecal and urinary samples from patients with CD and UC. Marchesi et al. [97] found that CD patients had lower fecal C2 and C4 levels, with CD patients exhibiting more pronounced SCFAs depletion than UC patients. Similarly, Stephens et al. [98] detected reduced C2 levels in urine samples from CD and UC patients, reinforcing the idea that SCFA metabolism is disrupted in IBD. Bjerrum et al. [99] extended these findings by showing that both CD and UC patients exhibited significant reductions in C3 and C4 in feces, emphasizing the potential role of microbial-derived SCFAs in gut barrier integrity and immune regulation. In a larger study, Lloyd-Price et al. [100] analyzed 132 IBD patients, identifying reduced C4 levels in IBD cases. Wang et al. [101] confirmed similar results, observing lower C2, C3, and C4 levels in CD and UC patients. However, not all studies support the depletion of SCFAs in IBD. Kiasat et al. [102] investigated plasma SCFA levels in 132 CD patients, 119 UC patients, and 205 controls, finding no significant differences between the groups. This suggests that SCFA alterations may be localized to the gut environment rather than being reflected in systemic circulation, indicating the need for tissue-specific assessments when evaluating SCFA dynamics in disease states.

However, despite their potential as markers of microbial activity and their ability to differentiate different types of enteropathies, there is still no established range that allows for an accurate diagnosis or the decision to institute dietary interventions or microbiota-modulating therapies that will allow for their recovery.

### 2.4. Indicators of Gut Functionality

The study of intestinal function based on stool composition is another group of methods that involves minimal invasion of the individual. Among these markers are those compounds that have not been absorbed, partly due to poor digestive function, and that appear in the feces. In this review, as we have indicated, we will make special mention of fatty acids and amino acids.

#### 2.4.1. Fatty Acids as Markers of Gut Health

The enterocyte, like other cells, is composed of a phospholipid-based bilayer that regulates membrane permeability to signaling molecules, metabolites, and ions, while maintaining structural integrity [103]. Although the specific role of phospholipids in the physiology of CIEs remains unclear, alterations in lipid metabolism have been identified as key factors in these conditions [103]. In fact, metabolomic studies have reported differences in lipid composition between healthy and diseased individuals [104]. Consequently, investigating lipid metabolism and fatty acid profiles in CIEs may provide valuable insights into disease pathophysiology and potential biomarkers for diagnosis and treatment monitoring. In this regard, changes in plasma phosphatidylcholine levels before and after dietary treatment in dogs diagnosed with FRE and IRE [105] have been identified.

Moreover, several studies have investigated alterations in lipid composition in both blood (Table 2) and fecal samples (Table 3), with a particular focus on long-chain fatty acids, including saturated (SFA), monounsaturated (MUFA), and polyunsaturated fatty acids (PUFAs). Crisi et al. [106] analyzed erythrocyte membrane phospholipids in dogs with FRE, IRE, and antibiotic-responsive enteropathy (ARE) compared to a control group and found a significant decrease in SFA such as palmitic acid (C16:0) and an increase in stearic acid (C18:0), which was attributed to altered elongase activity. However, despite evaluating different types of enteropathies (FRE, IRE, ARE), these authors did not observe significant changes in the lipid profiles among them. Similarly, Walker et al. [107] reported a reduction in serum SFA (C16:0, C18:0) in dogs with CIEs compared to controls, aligning with the findings of Crisi et al. [106]. However, in a subsequent study, Crisi et al. [108] did not find significant alterations in erythrocyte membrane SFA or MUFA proportions in cats with CIEs. The findings in human studies are largely consistent with those observed in dogs, with most studies reporting a decreased proportion of SFA in plasma or serum from patients with IBD, including CD and UC [109,110,111,112]. However, Murgia et al. [113] reported an increase in levels of C16:0 in the plasma of IBD patients compared to healthy controls.

The proportion of MUFA fatty acids in blood is also modified, although to varying degrees (Table 2). Galler et al. [114] reported lower plasma MUFA levels (C18:1) in Yorkshire terriers with CIEs, a finding consistent with those of Walker et al. [107] and Schwarz et al. [112] in humans. This aligns with the observed decrease in Δ9-desaturase activity in CIE-affected dogs, which impairs the conversion of SFA into their monounsaturated derivatives [106]. However, Wiese et al. [111] observed an increase in C18:1*n* − 9 in IBD patients, while Murgia et al. [113] found elevated palmitoleic acid (C16:1*n* − 7). It is important to consider that both MUFA and SFA originate not only from the dietary intake, but they can also be synthesized endogenously [115]. Their synthesis could be regulated, in addition to the presence of substrate availability (excess circulating carbohydrates for the synthesis of C16:0), enzymatic activity (Δ-9-desaturase to promote conversion of SFA to MUFA), and the availability of other fatty acids primarily involved in energy production or the synthesis of metabolites required for inflammatory processes [116].

Regarding PUFA content, most studies have reported decreased total PUFA levels, in the blood of companion animals with CIEs (Table 2). Walker et al. [107] observed a reduction in serum PUFA proportions, mainly linoleic acid (LA, *n* − 6) in CIE-affected dogs. Similarly, Crisi et al. [106] found lower erythrocyte PUFA C18:2*n* − 6 proportions, and a reduced *n* − 6/*n* − 3 ratio in dogs with CIEs; whereas long-chain PUFA (C20:3*n* − 3, C20:5*n* − 3, C22:6*n* − 3 and total *n* − 3) increased, accompanied by increased Δ6-desaturase and elongase activity. A later study in cats with FRE, IRE, or lymphoma reported similar findings [108]. Likewise, Higueras et al. [93] identified reduced total PUFAs and *n* − 3 (C20:5*n* − 3, C22:5*n* − 3) in the plasma of FRE dogs, alongside an increase in long-chain *n* − 6 PUFAs (C20:3*n* − 6). This fatty acid has been identified as the immediate precursor of prostaglandin E1 (PGE1) and arachidonic acid (C20:4*n* − 6), both involved in eicosanoid synthesis and the inflammatory regulation [117], as well as being components of the phospholipid membranes. In humans, increased long-chain PUFA proportions have been documented in patients with IBD (CD or UC) [110,111,112,113]. Moreover, some studies have reported an inverse correlation between circulating *n* − 6 fatty acids and disease severity [109,118,119]. However, other authors observed reduced plasma C20:3*n* − 6 proportions in dogs [114], decreased *n* − 3-PUFAs in patients with CD [120], or reductions in both long-chain *n* − 3 and *n* − 6 PUFA [119,121,122]. It is important to note that PUFAs come exclusively from dietary intake, and in some cases, such as long-chain PUFAs, can be synthesized through desaturation and elongation of their precursors, primary C18:2*n* − 6 and C18:3*n* − 3 [123]. Generally, PUFAs are preferentially utilized for energy production, while long-chain PUFAs are essential for synthesizing compounds involved in inflammation [116]. Therefore, discrepancies between studies may be attributed not only to diet composition or malabsorption, but also to the state of malnutrition, increased utilization of PUFA for inflammatory mediator synthesis, or their role in intestinal epithelial repair [124].

Concerning fecal lipid composition (Table 3), it reflects intestinal digestion, absorption, and bacterial metabolism of fatty acids; consequently, in CIEs, increased fecal lipid excretion is often indicative of malabsorption. Thus, Honneffer et al. [125] found increased C16:0 and C18:0 proportions in feces from dogs with chronic enteropathy. Similarly, Galler et al. [79] observed increased fecal SFA (C16:0, C18:0) in CIE-affected Yorkshire terriers, with levels decreasing post-treatment. Likewise, Cagnasso et al. [78] found higher fecal SFA levels in dogs with PLE, indicating a more severe malabsorptive phenotype. Limited data exist regarding fecal lipid variations between different CIE types. Higueras et al. [94] reported increased fecal C14:0 and C18:0 in IRE dogs compared to controls, while IRE dogs only showed increased C14:0 proportions compared to FRE dogs. Additionally, these authors observed a direct correlation between fecal SFA (C18:0) proportions and CIBDAI or the Purina fecal score. Conversely, Higueras et al. [93] noted decreased C15:0 SFA proportions in FRE dogs compared to controls. This odd-chain fatty acid (OCFA) originates not only from dietary intake but can also be endogenously synthesized from C3 [126]. Therefore, its reduced presence in feces may reflect lower microbiota production, as suggested by Higueras et al. [93], who found an inverse correlation between C15:0 and the CIBDAI index. In cats, Sung et al. [127] and Giordano et al. [128] both found increased fecal SFA (C14:0, C18:0). A similar pattern has been reported in human studies, where increased fecal SFA proportions, mainly C16:0 and C18:0, have been observed in patients with chronic intestinal diseases [129].

**Table 3:** Changes in the fecal fatty acid profile in dogs or cats with different chronic inflammatory enteropathies (CIEs).

Disease	Species	Sample	Findings	Reference
Chronic enteropathy (*n* = 15) Control (*n* = 15)	Dogs	Feces	CIEs: SFA: ↑ C16:0, ↑ C18:0, ↑ MUFA *n* − 9, PUFA: ↑ C22:3*n* − 3, ↑ AA, ↑ C22:2*n* − 6	[125]
Food-responsive enteropathy (*n* = 9) Control (*n* = 6)	Dogs	Feces	CIEs: ↓ C15:0, ↓ C16:1*n* − 9, ↓ C16:1*n* − 7, ↓ C20:5*n* − 3, ↓ MUFA, ↓ C16:1/C16:0, ↑ C18:0, ↑ elongase C16/C18	[93]
Inflammatory Intestinal Disease (*n*= 11) T-phenotype small cell lymphoma (*n*= 11) Control (*n*= 14)	Cats	Feces	IBD, lymphoma: ↑ long-chain fatty acids: PUFA, AA, DHA, ↑ MUFA *n* – 9. No differences between control vs. IBD.	[130]
Chronic enteropathy (active) (*n* = 14) Chronic enteropathy (*n* = 11) Control (*n* = 26)	Dogs (Yorkshire terrier)	Feces	CIE active: ↑ long fatty acids (↑ MUFA *n* − 9, ↑ LA, ↑ ALA). Reduction in them after treatment.	[79]
Chronic enteropathy (*n* = 56): Inflammatory Intestinal Disease (*n*= 22) T-phenotype small cell lymphoma (*n* = 34) Control (*n* = 77)	Cats	Feces	CIEs: SFA: ↑ C14:0, ↑ C18:0; MUFA: ↑ C18:1*n* − 9, ↑ C22:1*n* − 9, ↑ C20:1*n* − 9, ↑ C24:1*n* − 9; PUFA: ↑ ALA, AA	[127]
Protein-losing enteropathy (PLE) (*n*= 38) Control (*n*= 47)	Dogs	Feces	PLE: ↑ SFA (C16:0, C18:0); ↑ MUFA *n* − 9 (oleico); PUFA: ↑ LA, ↑ AA; ↑ total fatty acids. After treatment: ↓ MUFA *n* − 9 y ↓C18:0	[78]
Food-responsive enteropathy (*n* = 35) Immunosupressant-responsive enteropathy (*n* = 18) Control (*n* = 22)	Dogs	Feces	IRE vs. control: ↓ C16:1*n* − 7, ↓ C18:1*n* − 9, ↓ C18:2*n* − 6, ↓ C18:3*n* − 3, ↓ MUFA, ↓ PUFA, ↓ n − 6, ↓ C16:1*n* − 7/C16:0, ↓ C18:1*n* − 9/C18:0, ↑ elongase C18/C16, ↑ SFA:C14:0, C18:0; IRE vs. FRE: ↓ C16:1*n* − 7, ↓ PUFA, ↓ C16:1*n* − 7/C16:0, ↑ C14:0, ↑ C18:3*n* − 3, ↑ C22:5*n* − 3, ↑ SFA, ↑ *n* − 3, ↑ elongase C18/C16	[94]
Chronic enteropathy (*n* = 34): Food-responsive enteropathy (*n*= 13) Inflammatory Intestinal Disease (*n*= 15) T-phenotype small cell lymphoma (*n* = 6) Control (*n* = 27)	Cats	Feces	CIEs: SFA: ↑ C14:0, ↑ C18:0, ↑ C24:1*n* − 9 y ↑ C22:1*n* − 9 (MUFA *n* − 9), ↑ AA (PUFA); Significant changes in control vs. IBD + lymphoma: C14:0, C18:0, C24:1*n* − 9, AA. The FRE metabolome was more similar to controls than to IBD or lymphoma	[128]

CIEs: Chronic inflammatory enteropathies; FRE: Food-responsive enteropathy; IBD: Inflammatory Intestinal enteropathy; IRE: Immunosuppressant-responsive enteropathy; PLE: protein-losing enteropathy; SFA: sum of total saturated fatty acids; MUFA: sum of total monounsaturated fatty acids; PUFA: sum of total polyunsaturated fatty acids; AA: arachidonic acid (C20:4*n* − 6); DHA: docosahexanoic acid (C22:6*n* − 3); LA: linoleic acid (C18:2*n* − 6); ALA: linolenic acid (C18:3*n* − 3).

In relation to MUFA excretion in dogs with CIEs (Table 3), Honneffer et al. [125] observed increased fecal MUFA proportions. Similarly, Galler et al. [79] found elevated fecal MUFA (*n* − 9) levels in CIE-affected Yorkshire terriers, which normalized after treatment, indicating malabsorption. Cagnasso et al. [78] also found increased fecal MUFA, particularly oleic acid (*n* − 9), in dogs with PLE. Moreover, in cats, Marsilio et al. [130], Sung et al. [127], and Giordano et al. [128] all reported higher fecal MUFA (*n* − 9) in feline CIEs, indicating similar excretion patterns to those observed in dogs. However, Higueras et al. [93] noted a significant decrease in the proportion of C16:1*n* − 7 and C16:1*n* − 9 in the feces of dogs with FRE compared to controls. Similarly, in a subsequent, more comprehensive study evaluating different CIE types, Higueras et al. [94], found that the most marked reductions in C16:1*n* − 7 occurred in IRE dogs, followed by FRE dogs and control dogs. Thus, this fatty acid was proposed as a potential marker for differentiating enteropathy types. Additionally, the C16:1*n* − 7/C16:0 ratio, as an indicator of Δ-9-desaturase activity, was the lowest in IRE dogs. Moreover, these authors reported a negative correlation between Δ-9-desaturase activity and disease severity, as assessed by the CIBDAI. A decrease in the gene expression of ∆-9-desaturase has been described in humans with UC [131] and in mice [132], and a reduction of this enzyme has also been observed at the intestinal membrane due to gut damage [133]. The fact that altered desaturase activity was observed in dogs, but not in cats, highlights the importance of species-specific approaches in the diagnosis and treatment of CIE-related lipid metabolism disorders. Also, in human studies, discrepancies exist regarding fecal MUFA proportions in IBD. Some authors observed increased fecal MUFA in CD patients compared to healthy controls [129], while others reported decreases in both CD and UC patients relative to controls [134]. Generally, MUFAs are considered fatty acids with an anti-inflammatory character, with specific compounds such as palmitoleic acid (C16:1*n* − 7) playing a crucial role in attenuating inflammatory damage. This has been demonstrated in murine models, where palmitoleic acid modulated the IL-6 and TNF-α/NF-κB pathways, exerting protective effects on intestinal inflammation [135,136].

Concerning PUFA excretion in feces (Table 3), several studies have demonstrated increased PUFA levels in dogs with CIEs, suggesting malabsorption and/or membrane destruction. Honneffer et al. [125] found higher fecal PUFA proportions, particularly C22:3*n* − 3, arachidonic acid (AA) and docosahexaenoic acid (DHA, C22:6*n* − 3), in dogs with CIEs. Similarly, Galler et al. [79] reported increased fecal linoleic acid (LA, C18:2*n* − 6) and linolenic acid (ALA, C18:3*n* − 3) in CIE-affected dogs, with a reduction following treatment. In dogs with PLE, Cagnasso et al. [78] observed similar increases in PUFA excretion, particularly LA and AA. However, in the study of Higueras et al. [94] comparing different enteropathies, IRE dogs presented lower PUFA proportions in fecal samples compared to controls and FRE dogs, despite increased excretion of ALA and total *n* − 3. These authors also found an inverse relationship between total PUFA in feces and the CIBDAI, while disease severity correlated positively with Δ-6-desaturase (measured as C18:3*n* − 6/C18:2*n* − 6 ratio) and elongase indices. These results suggest that, in more severely affected dogs, a greater proportion of long-chain PUFAs derived from precursors such as LA were present in feces and gradually decreased in dogs with less severe disease. Various authors have found enhanced activity of Δ-6 desaturases and elongases indices that result in a greater production of long-chain PUFA in dogs [106] or humans with CD [137]. In cats affected with IBD or T-phenotype small cell lymphoma, an increased proportion of PUFA, mainly AA [127,128,130], has been observed when compared to healthy controls. In humans, an increased fecal proportion of PUFA has also been reported in CD patients. Thus, Jansson et al. [129] reported increased fecal LA, ALA, and AA, while Alghamdi et al. [138] noted increased proportions of C20:3 and C22:3 acids and AA. Comparative studies between CD and UC patients have generally reported similar trends, with increased fecal long-chain PUFA (particularly *n* − 3 PUFA) [139], or AA [140] in both diseases. Changes in these fecal PUFA profiles may be attributed not only to malabsorption due to intestinal inflammation, but also due to the epithelial membranes’ destruction [93] or the higher use of these fatty acids for the synthesis of compounds involved in the inflammation process, since these fatty acids are mediators of prostanoids, leukotrienes, lipoxins, etc. [116].

In summary, blood profiles show reduced levels of specific fatty acids and alterations in enzymatic indices, indicative of impaired synthesis or increased metabolic utilization. In contrast, fecal profiles often exhibit elevated total lipid content, suggesting malabsorption, along with selective reductions in fatty acids of microbial or host origin, which may reflect intestinal dysbiosis or compromised enzymatic activity within the gut.

#### 2.4.2. Amino Acids as Markers of Gut Health

**Figure 2:**
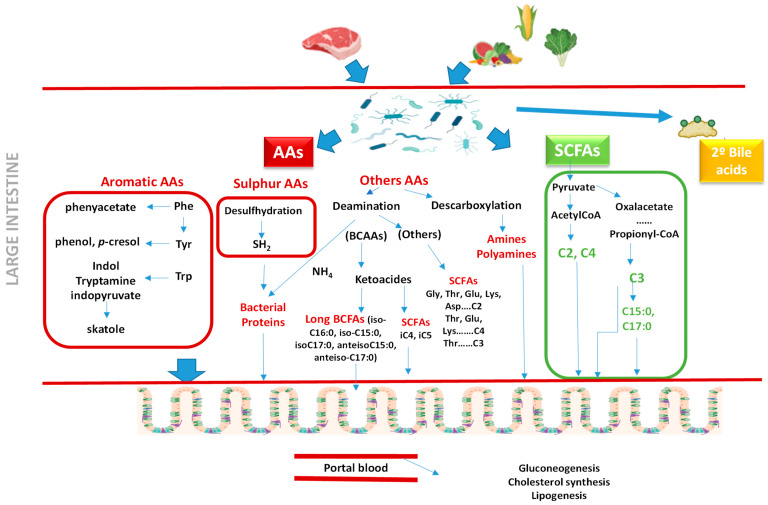
Synthesis of compounds by the action of the microbiota from undigested material in the large intestine (Aas: amino acids; Phe: phenylalanine; Tyr: tyrosine; Trp: tryptophan; BCFAs: branched-chain fatty acids; SCFAs: short-chain fatty acids; C2: acetic acid; C3: propionic acid; C4: butyric acid; iC4: isobutyric acid; iC5: isovaleric acid).

Regarding amino acids, these compounds are also part of biological membranes, along with lipids, and have important functions in the intestine [141]. Thus, some play a fundamental role in maintaining the integrity of the intestinal barrier, enabling epithelial renewal and mucin production, as well as maintaining tight protein junctions [142]. Moreover, some amino acids (such as glutathione formed from glutamate, cysteine, and glycine; or arginine) enhance immunity through optimizing cellular antioxidative responses and inhibiting inflammation [143]. In addition, amino acids (from diet or endogenous proteins) can serve as a source of carbon and nitrogen for microorganisms, encouraging them to produce beneficial substances such as SCFAs or provide amino acids to the host, contributing together to the individual’s homeostasis [144] (Figure 2).

However, within the mixture of metabolic compounds produced by the microbiota, there are also hydrogen sulfide (H_2_S), polyamines, phenols, indoles, ammonium (NH_4_), etc. that can exert beneficial or harmful effects depending on their concentration [145] (Figure 2). Consequently, the protein intake level and source and level of consumption of certain amino acids can affect the composition and metabolic activity of the intestinal microbiota and thus its fermentation products potentially available to the host [145]. Recent research has highlighted the role of amino acids as potential biomarkers for CIEs, with alterations observed in both blood and fecal samples. The variations in amino acid levels reflect underlying metabolic disruptions, including malabsorption, inflammation, and changes in microbial metabolism. This review discusses the key findings regarding amino acid changes in serum and fecal samples of dogs with CIEs, integrating evidence from different studies.

Alterations in amino acid metabolism have been documented in dogs and cats with CIEs, including IRE, PLE, and FRE. Several studies have highlighted these metabolic changes, providing insights into potential biomarkers and the pathophysiological mechanisms underlying these disorders (Table 4). Minamoto et al. [146] reported a general reduction in serum free amino acids in dogs with idiopathic IBD, including histidine, glutamine, tyrosine, tryptophan, cysteine, proline, and hydroxyproline, suggesting dysregulated amino acid metabolism that may contribute to chronic inflammation. Similarly, Tamura et al. [147] found decreased plasma levels of tryptophan, serine, methionine, and proline in dogs with IBD, with serine negatively correlated with the CCECAI. The observed reductions in essential and conditionally essential amino acids likely reflect malabsorption and increased utilization due to chronic inflammation. PLE in dogs has been specifically associated with decreased tryptophan levels, showing a significant correlation with albumin concentrations [148]. In dogs with FRE, [149] reported reductions in histidine, asparagine, glycine, cysteine, and leucine, along with a lower branched-chain amino acids/aromatic amino acids (BCAAs/AAAs) ratio compared to a healthy control. Human studies on CD or UC similarly reported reduced circulating amino acids [113,121,150,151,152,153,154]. However, other authors have reported amino acid-specific variations. A comprehensive study by Benvenuti et al. [155] analyzed the serum amino acid profile in dogs with IBD, identifying reductions in AAAs such as tyrosine, phenylalanine, and tryptophan, along with increases in serine, glutamic acid, arginine, threonine, proline, cystine, lysine, valine, and isoleucine compared to the control group. Notably, increased cystine levels suggested a compensatory mechanism or altered sulphur amino acid metabolism. Elevations in circulating amino acids involved in antioxidant and inflammatory responses, such as glycine, arginine, proline, methionine, and glutamate, have also been observed in CD patients [156,157,158], similar to the findings in dogs with CIEs [107,155]. These discrepancies between studies suggest that amino acid profiles may vary depending on the stage of the disease or the presence of malnutrition versus an active inflammatory response.

Regarding variations in BCAAs, Xu et al. [25] also reported increased free valine levels in dogs with CIEs, correlating with the CCECAI score, suggesting a potential link between amino metabolism and disease severity. Similarly, increased circulating BCAAs, such as isoleucine, valine, and leucine, have been observed in patients with CD or UC [156,157]. These amino acids play a crucial role in metabolism, contributing to glucose and lipid regulation and protein synthesis and overall intestinal health [159]. In addition, BCAAs can provide energy in the form of nitrogen for the synthesis of other amino acids and for cell division of enterocytes or immune cells proliferation [160]. However, as mentioned above, BCAA levels may also be reduced in circulation, due not only to poor absorption in the case of chronic inflammation but also increased metabolic utilization. It has been reported that BCAAs may contribute to the synthesis of fatty acids [161], including the production of SCFAs [93,162].

**Table 4:** Changes in the blood free amino acid profile in dogs with different chronic inflammatory enteropathies (CIEs).

Disease	Species	Sample	Findings	Reference
Inflammatory Intestinal Disease (*n* = 12) Control (*n* = 10)	Dogs	Serum	IBD: alteration in metabolism of amino acids (↓ histidine, ↓ glutamine, ↓ tyrosine, ↓ tryptophan, ↓ cysteine, ↓ proline, ↓ hydroxyproline)	[146]
Inflammatory Intestinal Disease (*n* = 15) Control (*n* = 10)	Dogs	Plasma	IBD: ↑ valine alanine correlated with CCECAI	[25]
Protein-losing enteropathy (*n* = 30) Control (*n* = 12)	Dogs	Serum	PLE: ↓ tryptophan correlation tryptophan and albumin	[148]
Inflammatory Intestinal Disease (*n* = 10) Control (*n* = 12)	Dogs	Plasma	IBD: ↓ tryptophan, ↓ serine, ↓ metionine, ↓ proline. Negative correlation between serine-CCECAI	[147]
Inflammatory Intestinal Disease (*n* = 51) Control (*n* = 26)	Dogs	Serum	IBD: ↓ tyrosine, ↓ phenylalanine, ↓ tryptophan (AAA); ↑ serine, ↑ glutamic acid, ↑ arginine, ↑ threonine, ↑ proline, ↑ cystine, ↑ lysine, ↑ valine, ↑ isoleucine	[155]
Chronic Enteropathy (*n* = 55) Control (*n* = 204)	Dogs	Serum	CIEs:↑ phenylalanine, ↓ glycine	[107]
Food-responsive enteropathy (*n* = 9) Control (*n* = 6)	Dogs	Plasma	Plasma FRE: ↓ histidine, ↓ asparagine, ↓ glycine, ↓ cystine, ↓ leucine, ↓ BCAA/AAA; ↑ phenylalanine	[149]
Chronic enteropathy (*n* = 8) Control (*n* = 16)	Cats	Plasma, urine	CIEs: Plasma: ↑ alanine, ↑ glutamine, ↑ valine, ↑ isoleucine, ↑ phenylalanine. Plasmatic metabolites (alanine, glutamine, betaine, glycerol) and urine as predictor of the response to diet.	[163]
Inflammatory Intestinal Disease (*n* = 13) T-phenotype small cell lymphoma (*n* = 13) Control (*n* = 14)	Cats	Serum	IBD, lymphoma: ↑ alanine, ↑ histidine, ↑ methionine, ↑ lysine, ↑ valine ↓ Metabolites derived from tryptophan; IBD vs. lymphoma: changes in tyrosine and other compounds	[164]

IBD: Inflammatory Intestinal enteropathy; FRE: Food-responsive enteropathy; IRE: Immunosuppressant-responsive enteropathy; PLE: protein-losing enteropathy; CIEs: Chronic inflammatory enteropathies.

Concerning AAAs, Walker et al. [107] investigated amino acid levels in dogs with CIEs and reported elevated plasma phenylalanine levels. Similarly, Walker et al. [106] and Higueras et al. [149] also observed an increase in plasma phenylalanine values in dogs, while Kathrani et al. [163] reported similar findings in cats, and Dawiskiba et al. [157] documented the same trend in patients with CD or UC. This increase is related to the fact that it has been observed that phenylalanine reflects the body protein breakdown [164], a process commonly exacerbated by malnutrition related to digestive disorders. Furthermore, some studies have found a positive correlation between elevated circulating phenylalanine and disease severity [149]. Additionally, a negative correlation was observed between the ratio of BCAAs/AAAs and disease severity [149], suggesting an imbalance in amino acid metabolism in affected individuals.

In cats, Kathrani et al. [163] investigated amino acid metabolism across different biological samples (plasma and urine) in animals with CIEs. They reported increased plasma concentrations of alanine, glutamine, valine, isoleucine, and phenylalanine, with metabolites such as alanine, glutamine, betaine, and glycerol also detected in urine. The findings suggest that urinary metabolites could serve as non-invasive markers for predicting dietary responses in CIEs. Furthermore, Questa et al. [165] analyzed amino acid profiles in cats with IBD and T-phenotype small cell lymphoma. Both diseases were associated with increased plasma concentrations of alanine, histidine, methionine, lysine, and valine. In contrast, tryptophan metabolism appeared to be altered, with differences in tyrosine and other compounds between IBD and lymphoma, indicating potential metabolic distinctions between inflammatory and neoplastic processes.

While serum amino acid alterations provide insights into systemic metabolic disruptions, fecal amino acid profiles offer a complementary perspective by reflecting changes in gut microbial metabolism and nutrient malabsorption (Table 5). However, studies analyzing fecal amino acid profile in companion animals remain scarce. In dogs diagnosed with CIEs, Honneffer et al. [125] reported increased fecal concentrations of cysteine, glycine, phenylalanine, valine, leucine, and lysine, as well as metabolites derived from tryptophan. Similarly, Pilla et al. [166] observed significantly elevated fecal levels of proline, valine, leucine, isoleucine, tyrosine, asparagine, aspartic acid, cysteine, cystine, glutamic acid, glycine, methionine, phenylalanine, serine, and threonine in dogs with IBD. These findings suggest that impaired intestinal absorption contributes to amino acid concentrations in the gut lumen. However, other studies have observed less pronounced alterations in fecal amino acid profiles, with increases in cysteine levels in dogs with FRE compared to control dogs [149]. Moreover, in a comparison of IRE and FRE dogs, Higueras et al. [162] found that dogs with IRE had increased tyrosine and threonine levels compared to the control group, whereas FRE dogs did not exhibit significant changes relative to IRE dogs. An increased presence of AAAs such as tyrosine in fecal matter may promote the formation of potentially toxic compounds to enterocytes like p-cresol [167] under certain intestinal conditions. Additionally, higher fecal threonine levels could be related to the preference of this amino acid by the cells of the intestinal mucosa for the synthesis of mucus [168].

In cats, Marsilio et al. [130] found that both IBD and T-cell lymphoma were associated with increased fecal aspartate, cysteine, phenylalanine, leucine, and valine, along with alterations in tryptophan metabolism. The similarity in amino acid disruptions between feline and canine CIEs suggests a shared metabolic disturbance in protein digestion and microbial amino acid metabolism across species. Consistently, increases in fecal amino acid concentrations have been reported in most studies in humans with chronic intestinal diseases [97,99,129,169,170,171], further supporting the relevance of amino acid dysregulation in gut pathophysiology.

In summary, consistent alterations in amino acid profiles have been observed in both the serum and feces of dogs with CIEs. These changes reflect malabsorption, inflammation, and microbial dysbiosis. Some amino acids, such as phenylalanine and branched-chain amino acids, correlate with disease severity. Fecal amino acid profiles complement serum findings and may serve as non-invasive biomarkers. Similar trends are seen in human IBD, supporting their relevance across species.

#### 2.4.3. Other Indicators of Gut Functionality

Cobalamin (vitamin B_12_) deficiency is common in dogs with CIEs. It is associated with alterations in the intestinal microbiome or impaired absorption [172,173,174]. Lower serum cobalamin concentrations have been shown to correlate with higher disease activity scores [59,175,176] and more severe ileal damage [177], as cobalamin is primarily absorbed in this portion of the intestine [173]. Therefore, hypocobalaminemia has been identified as a negative prognostic marker in dogs with CIEs [175,176,178]. Probiotic supplementation does not appear to improve their levels [179]; therefore, direct supplementation is recommended to alleviate clinical signs in more severe cases [8,180]. A similar situation is observed in cats, where vitamin B_12_ deficiency is prevalent in CIEs and where supplementation is advised. However, it should be noted that this is not a specific biomarker for this condition, as its levels can be altered by other processes and diseases [65,181,182,183,184].

The serum levels of folate (vitamin B_9_) in dogs with CIEs have been shown to vary, with levels ranging from increased to normal or decreased [25,59,185]. Hyperfolatemia has been observed in cases of dysbiosis [59,173,186,187,188,189]. Conversely, hypofolatemia may be attributable to proximal intestinal damage, as it is in the duodenum and proximal jejunum where this vitamin is absorbed [59,65]. This variability in its concentrations renders folate a less effective biomarker for canine CIEs than cobalamin [185].

## 3. Conclusions

The reviewed literature indicates that CIEs are associated with distinct alterations in both systemic and intestinal biomarkers. These alterations include changes in lipid metabolism (e.g., shifts in saturated, monounsaturated, and polyunsaturated fatty acids), amino acid profiles, and inflammatory markers. Together, these biomarkers reflect not only the severity of mucosal inflammation but also disruptions in digestion, absorption, and the microbial metabolism. Systemic markers (derived from blood, plasma, and erythrocyte membranes) consistently reveal a trend toward depletion of essential fatty acids and altered enzyme activities (e.g., reduced desaturase activity in dogs with CIEs). In contrast, fecal biomarkers often indicate increased excretion of specific lipid fractions and amino acids, likely reflecting malabsorption and localized mucosal dysfunction. This dichotomy between systemic and fecal markers underscores the importance of a combined diagnostic approach.

Understanding the metabolic alterations in fatty acids and amino acids offers promising avenues for therapeutic intervention. Nutritional strategies aimed at restoring SCFA production (e.g., dietary fiber supplementation or probiotics) and correcting amino acid imbalances may help reestablish intestinal barrier function and modulate the inflammatory response. Future studies should focus on the standardization of biomarker assays across species and validate integrated biomarker panels for improved clinical management of CIEs. Given the multifactorial nature of CIEs, the use of a single biomarker is unlikely to provide sufficient diagnostic accuracy. Instead, a panel of biomarkers that includes lipid profiles, amino acid patterns, and inflammatory mediators offers a more comprehensive view of gastrointestinal functionality and disease status. Such integrated panels may improve the differentiation between various CIE types and enhance the monitoring of treatment responses. Future research should also focus on standardizing assay protocols, establishing reference ranges across species, and validating integrated biomarker panels in large, multicenter studies. This will be essential to translate these findings into clinical practice and improve outcomes for animals suffering from CIEs.

## Figures and Tables

**Table 1:** Changes in short-chain fatty acids (SCFAs) in dogs with different chronic inflammatory enteropathies (CIEs).

Disease	Species	Sample	Findings	Reference
Inflammatory Intestinal Disease (*n* = 15) Control (*n* = 10)	Dogs	Feces	No differences in SCFAs between groups	[25]
Chronic enteropathy (*n* = 73) Control (*n* = 49)	Dogs	Feces	CIEs: ↓ C2, C3, ∑SCFAs	[76]
Food-responsive enteropathy (*n* = 9) Control (*n* = 6)	Dogs	Feces	FRE: ↓ C2, C3, iC4, iC5, ∑SCFAs	[93]
Inflammatory Intestinal Disease (*n* = 6) Control (*n*= 16)	Dogs	Feces	IBD: ↓ C2, C3	[57]
CIEs: Inflammatory Intestinal Disease (*n*= 6) T-phenotype small cell lymphoma (*n* = 6) Unidentified enteropathy (*n* = 3) Control (*n* = 13)	Cats	Feces	CIEs: ↓ C3, iC4, ↑ C4 and ∑SCFAs	[95]
Food-responsive enteropathy (*n* = 35) Immunosuppressant-enteropathy (*n* = 18) Control (*n* = 22)	Dogs	Feces	IRE vs. control: ↓ C2, C3, ∑SCFAs	[94]

CIEs: Chronic inflammatory enteropathies; FRE: Food-responsive enteropathy; IBD: Inflammatory Intestinal Disease; IRE: Immunosuppressant-responsive enteropathy; C2: acetic acid; C3: propionic acid; C4: butyric acid; ∑SCFAs: sum of total short-chain fatty acids; iC4: isobutyric acid; iC5: isovaleric acid.

**Table 2:** Changes in the blood fatty acid profile in dogs or cats with different chronic inflammatory enteropathies (CIEs).

Disease	Species	Sample	Findings	Reference
Inflammatory Intestinal Disease (*n* = 12) Control (*n* = 10)	Dogs	Serum	IBD: no differences in lipid metabolism with controls.	[96]
Food-responsive enteropathy (*n* = 16) Immunosuppressants-responsive enteropathy (*n*= 16)	Dogs	Blood and plasma phospholipids	Differences in phospholipids between both groups. Phosphatidylcholine changed from PC38:4 before treatment to lysophosphatidylcholine 18:0 after treatment.	[105]
Chronic enteropathy (*n* = 48): Food-responsive enteropathy (*n* = 28) Antibiotic-responsive enteropathy (*n* = 5) Immunosuppressants-responsive enteropathy (*n* = 15) Control (*n* = 68)	Dogs	Erythrocyte membrane phospholipids	CIEs: SFA:↓ C16:0, ↑ C18:0; PUFA: ↓ LA, ↑ C20:3*n* − 6, ↑ EPA, ↑ DHA, ↑ *n* − 3, ↓ SFA/MUFA, ↓ *n* − 6/*n* − 3, ↑ elongase (C18/C16), Δ6-desaturase, ↓ Δ5-desaturase, ↓ Δ9-desaturase. No statistical differences between CE groups.	[106]
Food-responsive enteropathy (*n* = 9) Control (*n* = 6)	Dogs	Plasma	FRE: ↑ C20:3n − 6, ↓ C20:5*n* − 3, ↓ C22:5*n* − 3, ↓ ∑PUFA	[93]
Chronic enteropathy (*n* = 13) Control (*n* = 20)	Dogs (Yorkshire terrier)	Plasma	CIEs: ↓ C18:1, ↓ PUFA (↓ C20:2*n* − 6, ↓ C20:3*n* − 6)	[114]
Chronic enteropathy (*n* = 55) Control (*n* = 204)	Dogs	Serum	CIEs: ↓ SFA, ↓ C16:0, ↓ C18:1, ↓ PUFA, (LA, *n* − 6)	[107]
Chronic enteropathy (*n* = 41): Food-responsive enteropathy (*n* = 17) Inflammatory Intestinal Disease (*n* = 15) T-phenotype small cell lymphoma (*n* = 9) Control (*n* = 43)	Cats	Erythrocyte membrane phospholipids	CIEs: PUFA: ↑ C22:5*n* − 3, ↑ DHA, ↑ *n* − 3 PUFA; ↓ LA, ratio *n* − 6/*n* − 3, ↑ Δ6-desaturase No changes between CE groups.	[108]

IBD: Inflammatory Intestinal Disease; CIEs: Chronic inflammatory enteropathies; FRE: Food-responsive enteropathy; IRE: Immunosuppressant-responsive enteropathy; PLE: protein-losing enteropathy; SFA: sum of total saturated fatty acids; MUFA: sum of total monounsaturated fatty acids; PUFA: sum of total polyunsaturated fatty acids.

**Table 5:** Changes in the fecal amino acid profile in dogs or cats with different chronic inflammatory enteropathies (CIEs).

Disease	Species	Sample	Findings	Reference
Chronic enteropathy (*n* = 15) Control (*n* = 15)	Dogs	Feces	CIEs: ↑ cisteine, ↑ glycine, ↑ phenylalanine, ↑ valine, ↑ leucine, ↑ lysine ↓ metabolites derived from tryptophan	[125]
Inflammatory Intestinal Disease (*n* = 9) Control (*n* = 13)	Dogs	Feces	IBD: ↑ proline, ↑ valine, ↑ leucine, ↑ isoleucine, ↑ alanine, ↑ tryptophan, ↑ asparagine, ↑ aspartic acid, ↑ cisteine, ↑ cystine, ↑ glutamic acid, ↑ glycine, ↑ methionine, ↑ phenylalanine, ↑ serine, ↑ threonine	[166]
Food-responsive enteropathy (*n* = 9) Control (*n* = 6)	Dogs	Feces	FRE: ↓ phenylalanine, ↑ cystine,	[149]
Food-responsive enteropathy (*n* = 35) Immunosuppressant-responsive enteropathy (*n* = 18) Control (*n* = 22)	Dogs	Feces	IRE vs. control: ↑ Tyrosine, ↑ Threonine; FRE vs. control: ↑ Tyrosine; FRE vs. IRE: no changes	[162]
Inflammatory Intestinal Disease (*n* = 11) T-phenotype small cell lymphoma (*n* = 11) Control (*n* = 14)	Cats	Feces	IBD, lymphoma: ↑ aspartate, ↑ cisteine, ↑ phenylalanine, ↑ leucine, ↑ valine ↓ Metabolites derived from tryptophan Alteration in amino acid metabolism	[130]

IBD: Inflammatory intestinal disease; FRE: Food-responsive enteropathy; IRE: Immunosuppressant-responsive enteropathy; PLE: protein-losing enteropathy; CIEs: Chronic inflammatory enteropathies.

## Data Availability

Data are contained within the article.

## Abbreviations

The following abbreviations are used in this manuscript:

CIEs	Chronic Inflammatory Enteropathies
IBD	Inflammatory Bowel Disease
FRE	Food-Responsive Enteropathy
IRE	Immunosuppressant-Responsive Enteropathy
PLE	Protein-Losing Enteropathy
SCFAs	Short-Chain Fatty Acids
SAT	Saturated Fatty Acids
MUFA	Monounsaturated Fatty Acids
PUFA	Polyunsaturated Fatty Acids
LA	Linoleic Acid (C18:2*n* − 6)
ALA	Alpha-Linolenic Acid (C18:3*n* − 3)
EPA	Eicosapentaenoic Acid (C20:5*n* − 3)
DHA	Docosahexaenoic Acid (C22:6*n* − 3)
AA	Arachidonic Acid (C20:4*n* − 6)
C2	Acetic Acid
C3	Propionic Acid
C4	Butyric Acid
iC4	Isobutyric Acid
iC5	Isovaleric Acid
CCECAI	Canine Chronic Enteropathy Clinical Activity Index
CIBDAI	Canine Inflammatory Bowel Disease Activity Index
FABP	Fatty Acid Binding Protein
I-FABP	Intestinal Fatty Acid Binding Protein
IAP	Intestinal Alkaline Phosphatase
L/M	Lactulose/Mannitol Ratio
LPS	Lipopolysaccharide
MPO	Myeloperoxidase
pANCA	Perinuclear Anti-Neutrophilic Cytoplasmic Antibodies
CRP	C-Reactive Protein
IgA	Immunoglobulin A
DI	Dysbiosis Index
GPCR	G Protein-Coupled Receptors
BCAAs	Branched-Chain Amino Acids
AAAs	Aromatic Amino Acids
PC	Phosphatidylcholine
SII	Systemic Immune-Inflammation Index
PLR	Platelet-to-Lymphocyte Ratio
NLR	Neutrophil-to-Lymphocyte Ratio
OCFA	Odd-Chain Fatty Acids
